# Dynamics of Bacterial and Fungal Communities during the Outbreak and Decline of an Algal Bloom in a Drinking Water Reservoir

**DOI:** 10.3390/ijerph15020361

**Published:** 2018-02-18

**Authors:** Haihan Zhang, Jingyu Jia, Shengnan Chen, Tinglin Huang, Yue Wang, Zhenfang Zhao, Ji Feng, Huiyan Hao, Sulin Li, Xinxin Ma

**Affiliations:** 1 School of Environmental and Municipal Engineering, Xi’an University of Architecture and Technology, Xi’an 710055, Shaanxi Province, China; jiajingyuxiann@163.com (J.J.); chenshengnan@xauat.edu.cn (S.C.); huangtinglin@xauat.edu.cn (T.H.); yuewang_17@163.com (Y.W.); zhenfangzhao@163.com (Z.Z.); fengji0423@163.com (J.F.); JiandaHuiyanHe@163.com (H.H.); SulinLiSX@163.com (S.L.); mxxstudy@163.com (X.M.); 2 Institute of Environmental Microbial Technology, Xi’an University of Architecture and Technology, Xi’an 710055, Shaanxi Province, China

**Keywords:** drinking water reservoir, algal bloom, water fungal community composition, high-throughput sequencing

## Abstract

The microbial communities associated with algal blooms play a pivotal role in organic carbon, nitrogen and phosphorus cycling in freshwater ecosystems. However, there have been few studies focused on unveiling the dynamics of bacterial and fungal communities during the outbreak and decline of algal blooms in drinking water reservoirs. To address this issue, the compositions of bacterial and fungal communities were assessed in the Zhoucun drinking water reservoir using 16S rRNA and internal transcribed spacer (ITS) gene Illumina MiSeq sequencing techniques. The results showed the algal bloom was dominated by *Synechococcus*, *Microcystis*, and *Prochlorothrix*. The bloom was characterized by a steady decrease of total phosphorus (TP) from the outbreak to the decline period (*p* < 0.05) while Fe concentration increased sharply during the decline period (*p* < 0.05). The highest algal biomass and cell concentrations observed during the bloom were 51.7 mg/L and 1.9×10^8^ cell/L, respectively. The cell concentration was positively correlated with COD_Mn_ (*r* = 0.89, *p* = 0.02). Illumina Miseq sequencing showed that algal bloom altered the water bacterial and fungal community structure. During the bloom, the dominant bacterial genus were *Acinetobacter* sp., *Limnobacter* sp., *Synechococcus* sp., and *Roseomonas* sp. The relative size of the fungal community also changed with algal bloom and its composition mainly contained Ascomycota, Basidiomycota and Chytridiomycota. Heat map profiling indicated that algal bloom had a more consistent effect upon fungal communities at genus level. Redundancy analysis (RDA) also demonstrated that the structure of water bacterial communities was significantly correlated to conductivity and ammonia nitrogen. Meanwhile, water temperature, Fe and ammonia nitrogen drive the dynamics of water fungal communities. The results from this work suggested that water bacterial and fungal communities changed significantly during the outbreak and decline of algal bloom in Zhoucun drinking water reservoir. Our study highlights the potential role of microbial diversity as a driving force for the algal bloom and biogeochemical cycling of reservoir ecology.

## 1. Introduction

Algal blooms have become a worldwide and challenging water pollution problem in freshwater ecosystems due to harmful algae that cause a gradual degeneration of the water’s self-purification function and decrease the source water quality [1,2]. In the past several decades, a growing number of studies concerning the environmental factors of algal bloom outbreaks and decline have been explored [2]. Excessive exogenous nitrogen (N) and phosphorous (P), high temperature, and adequate light intensity have been identified as major abiotic triggers [3]. Furthermore, aquatic microbe such as bacterial and fungal communities also play a vital role in regulating water quality and the compositions of other organisms [4]. However, we still have limited understanding of the biotic factors, such as the interaction between algal bloom and the water microbial community composition.

Massive literatures reveal that most algal bloom studies have focused on lakes [5,6,7] and oceans [8]. Characterization of bacterial communities associated with algal blooms in eutrophic lakes from the United States [5], Norway [6], Germany [7] and China [8], investigated using the 16S rRNA gene sequence method, have demonstrated that algal blooms are a biological disturbance to lake bacterial communities. However, Scherer et al. [7] recently examined the dynamics of the water microbial community associated with cyanobacteria blooms in two recreational lakes in southern Germany and found that bloom toxicity could influence but did not change the bacterial community composition. The Actinobacteria group, in particular, remained rather stable during periods of higher algal cell concentration [7]. Berry et al. [5] also found that several bacterial species recovered quickly after the cyanobacterial harmful algal bloom in the western Lake Erie, suggesting that bacterial communities maybe somewhat resilient to algal blooms impacts [9]. Therefore, studies investigating bacterial community responses to algal blooms have offered conflicting results and more research is needed to fully understand the water microbial community during algal bloom events, especially in freshwater bodies like reservoirs.

Drinking water reservoirs have multiple functions including source water supply for human consumption and agriculture irrigation, and are especially important in arid and semiarid areas [10]. Water microbial communities play an essential role in reservoir ecosystems, mediating nutrient cycling and decomposition processes, and regulating water quality [11,12]. Compared with bacteria, fungi are key players as primary producers and food chain drivers in reservoirs [12,13]. Several aquatic fungi have the potential ability to inhibit harmful algae in freshwater ecosystems [14]. During the past few decades, the influence of algal blooms upon the water fungal community has not been extensively explored, as most studies focused on the overall bacterial community [5,6,7,8]. Although these studies indicate algal blooms may be an important factor shaping water microbial composition, the shifts in water microbial community structure are not well understood.

To this end, the main objective of the current study was to investigate the dynamics of water bacterial and fungal communities associated with algal blooms in the Zhoucun drinking water reservoir. The specific aims were to: (1) determine the physiochemical parameters of water quality during the phytoplankton bloom, (2) investigate the characteristics of shifts in water bacterial and fungal community diversity using high-throughput sequencing techniques during outbreak and decline of the algal bloom, and (3) assess the relationship among water quality, algal bloom and water microbial community compositions. This study will provide mechanisms of water microbial community structural and compositional responses to algal blooms in drinking water reservoir ecosystems that could serve as working hypotheses to be explored algal bloom control in other reservoir ecosystems.

## 2. Experimental Section

### 2.1. Study Site and Sample Collection

The field work was conducted at the Zhoucun drinking water reservoir (34°56′N, 117°40′E), located in Zaozhuang City, Shandong Province, in eastern China. Over the past few decades, a shortage of water has been a critical challenge for the development of Zaozhuang City. The Zhoucun drinking water reservoir is an important water source for Zaozhuang’s citizens [11,12]. The drainage area of the reservoir is 121 km^2^, and the total storage capacity is about 84 million m^3^. The maximum depth is 18 m, and the average depth is 13 m. Due to higher nitrogen and phosphorus concentrations, algal blooms mainly occur during summer seasons (from July to September), and more severe *Microcystis* blooms have occurred in recent years [15,16].

To further explore the dynamics of water microbial compositions during algal blooms in this reservoir, three sampling sites were selected, and marked as site A (34°56'38''N; 117°41'14''E), site B (34°56'38''N; 117°41'4''E), and site C (34°56'44''N; 117°41'14''E) and sampled from July to September, 2012. Samples were collected before the bloom period (13 July, 31 July, 16 August), during the bloom outbreak period (1 September), and during the bloom decline period (15 September and 30 September). At each sampling site, surface water (3.0 L, 0.5 m depth) was collected, stored in a sterile polyethylene bottle (Kangwei, Beijing, China), put in a cooler (8 ℃) and transferred to the laboratory within 24 h. One liter was used for water chemical parameters determination, and two liters were used for algal biomass and water microbial community diversity examination. Filter membranes for water microbial DNA extraction were directly stored at −20 ℃.

### 2.2. Water Physical and Chemical Parameters Measurement

To explore the water quality parameters, a multi-parameter water quality analyzer (Hydrolab DS5, Hach, Colorado, USA) was employed to measure the water temperature, dissolved oxygen concentration (DO), pH, turbidity, and electrical conductivity *in situ* [15]. In the laboratory, permanganate index (COD_Mn_), total nitrogen (TN), nitrate nitrogen (NO_3_^−^-N), nitrite nitrogen (NO_2_^−^-N), ammonia nitrogen (NH_4_^+^-N), total phosphorus (TP), iron (Fe), and manganese (Mn) concentrations were determine using standard methods previously described [15,17]. Briefly, TN and TP were measured by ultraviolet spectrophotometry (DR6000, Hach) after digestion (121 ℃, 30 min) of the water samples [17]. NO_3_^−^-N, NO_2_^−^-N, and NH_4_^+^-N concentrations were measured by a Flow Injection Analyzer (FIA, Seal Analytical AA3, Norderstedt, Germany). Permanganate index (COD_Mn_) was examined using a spectrophotometer (UV-mini 1240, Shimadzu, Kyoto, Japan). Fe and Mn were measured by atomic absorption spectrometry (FAAS, AA 6800, Shimadzu) as described previously [16]. The assays were performed in triplicate (*n* = 3).

### 2.3. Algal Cell Concentration and Biomass Determination

To determine the algal cell (AC) concentrations, 500 mL of the sampled water was filtered through a 0.45 μm polycarbonate membrane (47 mm diameter, Millipore, USA). Algal cell concentrations were counted microscopically (Olympus BX51, Tokyo, Japan) [18] using 100 μL of concentrated algal solution and reported in terms of ×10^4^ cells per liter. To examine the algal biomass (AB), the method described by Parulekar et al. [6] was performed with slight modifications. The algal cell enumeration and estimation of total volume was performed using a Utermöhl inverted microscope method (10 mL chamber). Microscopic examination (BX51, Olympus) was conducted as previously described [15]. The assays were performed in triplicate (*n* = 3).

### 2.4. Water Microbial DNA Extraction

To obtain the total community genomic DNA, 500 mL reservoir water was filtered by polycarbonate membrane (0.22 μm) (Millipore) in a clean bench (Jiangsu, China) [4]. Water microbial DNA was extracted and purified using a Water DNA Kit (Omega Bio-tek, CA, USA) following the manufacturer’s protocol. The genomic DNA concentration and purity were measured by a NanoDrop ND-2000 spectrophotometer (Thermo Scientific, Waltham, MA, USA) [6]. The purified DNA samples were stored at −80 ℃ for subsequent analyses [8].

### 2.5. Illumina MiSeq Sequencing and Sequence Analysis 

The Illumina Miseq sequencing technique was performed to evaluate the water bacterial and fungal communities. The 16S rRNA V4-V5 region, a hypervariable area of the 16S rRNA gene, was used for the water bacterial community analysis with bacteria-specific primers 515F and 907R [19,20]. The nuclear ribosomal internal transcribed spacer-1 (ITS1) was employed for the water fungal community analysis with fungi-specific primers 1737F and 2043R [21]. Primers were tagged with an adapter and a unique 6-bp error-correcting barcode sequence was added to 5' end of the reverse primer for the subsequent identification [19]. Amplification of bacterial 16S rRNA and fungal ITS1 regions were carried out using a PCR thermal cycler (ABI GeneAmp^®^ 9700, CA, USA) with 20 μL reaction volume mixture containing 10 ng of template DNA, 10 μM of the forward and reverse primers (0.4 μL), 5×reaction buffer (4 μL), 2.5 mM dNTPs (2 μL), Fast Pfu DNA polymerase (0.4 μL), and a balance of ddH_2_O. Detailed primer sets and PCR program process information are listed in Table 1. All of the PCR reactions were performed in triplicate (*n* = 3).

The PCR products were purified using a High Pure PCR product purification kit (Omega, Bio-tek) according to the protocol. PCR product concentration was quantified on an Agilent Bioanalyzer 2100 (Agilent Technologies, CA, USA). Paired-end sequencing (2 × 300 bp) was performed on an Illumina MiSeq platform at Shanghai Majorbio Bio-Pharm Technology (MBPT) Co., Ltd. (Shanghai, China).

### 2.6. Nucleotide Sequence Accession Number

Illumina MiSeq DNA sequence data were deposited in National Center for Biotechnology Information-Sequence Read Archive (NCBI-SRA) (http://www.ncbi.nlm.nih.gov/) database under the accession numbers SRP 041196 and SRP 041234 for bacterial and fungal communities, respectively.

### 2.7. Statistical Analysis

To compare the mean value of water quality parameters, algal cell concentration and algal biomass, statistical analyses were performed using one-way variance (ANOVA) followed by a Tukey HSD post-hoc test using SPSS (version 17.0, SPSS Inc, Chicago, IL, USA). The significance level was set at α = 0.05.

The Illumina MiSeq DNA sequences were determined with the Quantitative Insights Into Microbial Ecology (QIIME, version 1.9.1) software [22]. Water bacterial and fungal community sequence reads were first filtered by the QIIME pipeline [22]. The quality-trimmer removed reads are shorter than 50  bp [23]. After quality filtering and chimera removal, the abundance-based coverage estimator (ACE) index, *Chao* richness estimator (*Chao*1), Shannon diversity (*H*') and Simpson diversity (1/*D*) indices were calculated by the MOTHUR package (version 1.22.2 http://www.mothur.org) using Operational Taxonomic Units (OTUs) grouped at 0.97 level [24]. Water bacterial and fungal taxonomic assignments were performed using the Ribosomal Database Project (RDP) classifier (RDP Release 11.5, https://rdp.cme.msu.edu/), UNITE database (http://unite.ut.ee), and NCBI Taxonomy Browser [19,22,25]. Heat map profiles were performed using *R* software (version 3.2.3) [6,8]. Redundancy analysis (RDA) was conducted to reveal the water quality variables that correlated to changes in the structures of water microbial communities using the CANOCO software (version 4.5, Wageningen, The Netherlands). All data were log ^(x+1)^ transformed, and the water quality parameters best revealing the most influential gradients in the water bacterial and fungal community structure were assessed with Monte Carlo permutation test (*p* < 0.05) [11,26].

## 3. Results and Discussion

### 3.1. Water Quality Parameters

Algal blooms were found to have a significant impact on the water quality of the Zhoucun drinking water reservoir. The physical parameters (e.g., water temperature, DO, pH, turbidity, conductivity) and nutrient concentrations (e.g. COD_Mn_, TN, NO_3_^−^-N, NO_2_^−^-N, NH_4_^+^-N, TP, Fe and Mn) of each sample are shown in Table 2. Water quality characteristics were changed significantly during algal bloom outbreaks and decline from July to September, 2012 (Table 2). The highest temperature was observed on Jul. 31. Higher temperature can improve the growth of algae, and regulate the algal blooms [27]. The DO concentration and turbidity increased dramatically during the algal bloom (*p* < 0.01), while a higher conductivity was observed during the decline phase. The pH was observed to decrease significantly at the end of decline period (*p* < 0.01), which may be due to the fact that specific algal species can produce domoic acid [28]. The highest COD_Mn_ concentration was found during the outbreak on Sep. 1 and was more than twice as high as the COD_Mn_ observed in the decline period. A previous study conducted by Luria et al. [29] also found that dissolved organic matter can drive the growth of algae. The changes in water nutrient concentrations during algal bloom peak and decline shows that algal blooms influence the water quality in this reservoir. The lowest TN and NO_3_^-^-N concentrations were observed before algal bloom outbreak (*p* < 0.05, *p* < 0.01). However, the TP concentration decreased steadily throughout the algal bloom outbreak and decline periods. This result is consistent with the report by Bertain et al. [4], demonstrating that total phosphorus was an important driver of algal bloom intensity in western Lake Erie. The lowest concentration of Fe was observed before the bloom (0.05 mg/L on Jul. 13) and increased sharply through the algal bloom decline (*p* < 0.001). The study conducted by Landa et al. [30] determined the diverse bacterial responses to iron-induced phytoplankton blooms, and observed the contradictory results compared to the current finding.

The results from the present work are also consistent with the microcosms experiment conducted by Zhao et al. [31], demonstrating that water pH and dissolved organic carbon (DOC) showed significant changes during *Microcystis* decomposition. Additionally, the decomposition of *Microcystis* could drive the dynamics of bacterial community in the waters of Lake Taihu, in South China. Thus, algal blooms have the great potential to change water nutrient cycling of freshwater ecosystems like this reservoir.

### 3.2. Algal Biomass and Cell Concentration

**Figure 1:**
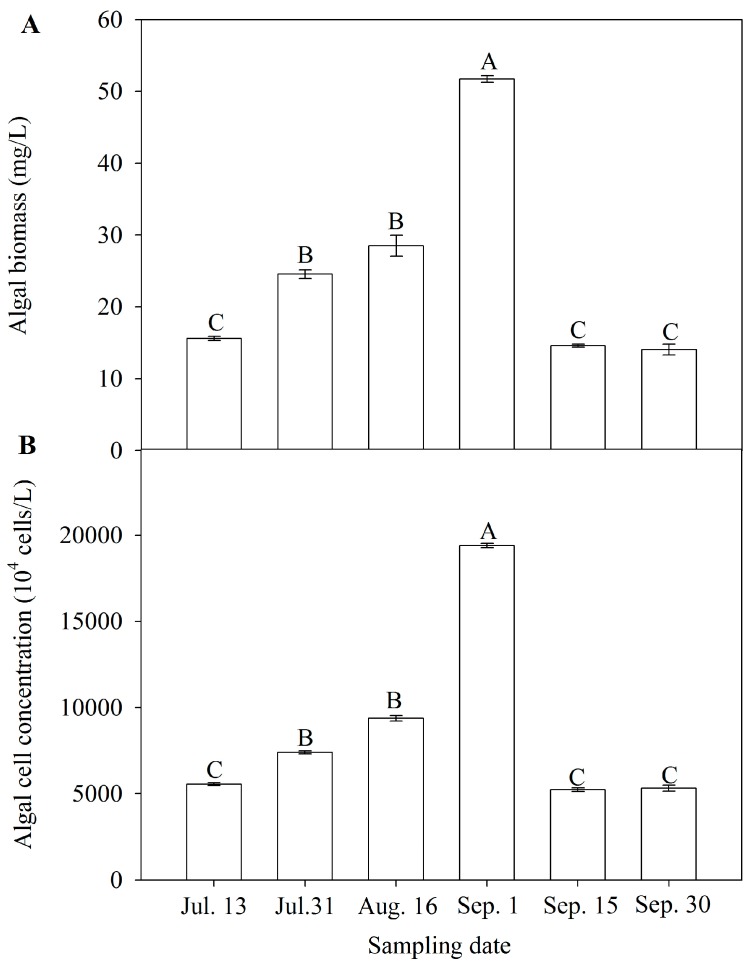
Algal biomass (**A**) and algal cell concentration (**B**) in Zhoucun drinking water reservoir during algal bloom outbreak and decline from July to September, 2012. Bars with different upper letter are significantly different at 0.01 levels. Error bars represent standard deviations (*n* = 3).

Variations in algal biomass and cell concentrations during algal bloom outbreak and decline (from July to September, 2012) are shown in Figure 1. The peaks of algal biomass (51.7 mg/L) and cell concentrations (1.9 × 10^8^ cell/L) appeared on Sep. 1 during the bloom (*p* < 0.01). Before the bloom, algal cell concentrations increased from 5.5 × 10^7^ cell/L on Jul. 13 to 9.4 × 10^7^ cell/L on Aug. 16 (*p* < 0.01). However, during the decline period, the cell concentration decreased from 5.3 × 10^7^ cell/L to 5.2 × 10^7^ cell/L (*p* > 0.05)( Figure 1B). A similar trend was also observed in algal biomass (Figure 1A). The cell concentration has a significant positive correlation with COD_Mn_ (*r* = 0.89, *p* = 0.02). Based on the microscopic analysis, the algal bloom was dominated by Cyanobacteria including *Synechococcus*, *Microcystis*, and *Prochlorothrix*. The result is consistent with a previous study [15]. In the cyanobacterial bloom that appeared in the summer of 2013 in Zhoucun drinking water reservoir, the algal cell concentration ranged from 7.3 × 10^7^ cell/L to 1.2 × 10^6^ cell/L. *Microcystis* spp., *Synechococcus* spp., and *Oscillatoria* sp. accounted for more than 50% of the algal biomass in that summer [15].

### 3.3. Water Bacterial and Fungal Communities

**Figure 2:**
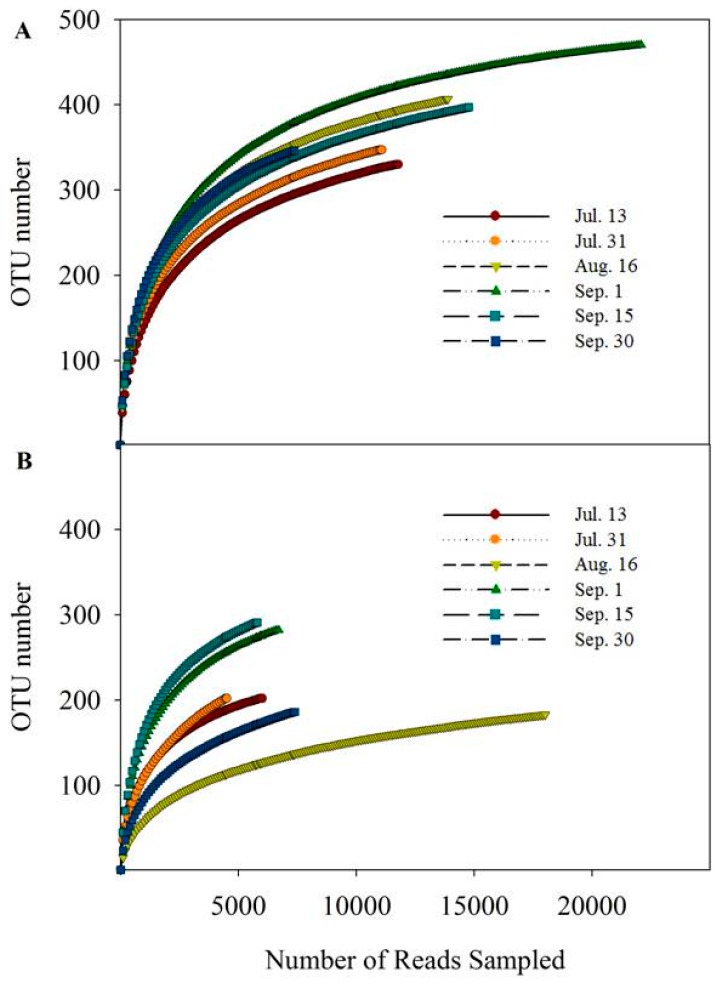
Waterbacterial (**A**) and fungal (**B**) communities operational taxonomic units (OTUs) number at 0.97 level and the reads number sampled in Zhoucun drinking water reservoir during algal bloom outbreak and decline from July to September, 2012.

Algal blooms have been widely investigated in reservoir ecosystems, but the response of water microbial communities to blooms has not been well characterized. In the current work, to explore the diversity and dynamics of water microbial community, high-throughput sequencing was carried out on an Illumina MiSeq platform. For the bacterial community, a total of 97,560 reads were recovered after chimeras removals and had an average length of 396 bp. For the fungal community, 48,119 reads were obtained with an average length of 320 bp. Bacterial and fungal diversity based on number of OTUs was evaluated (Figure 2). Rarefaction curves were generated from samples in Zhoucun drinking water reservoir during the algal bloom outbreak and decline. A total of 2,297 and 1,346 OTUs were generated for bacterial and fungal communities with a 97% match of the 16 S rRNA and ITS genes, respectively (Figure 2).

Algal bloom outbreaks often cause significant changes in water microbial communities. In this work, based on the Illumina MiSeq sequencing data, the diversity indices were calculated to explore the dynamic of water bacterial community structures. As shown in Table 3, the highest ACE index of 507 and *Chao* 1 of index 514 were observed during the algal bloom outbreak on Sep. 1. During the decline of the bloom (Sep. 15 and Sep. 30), ACE index and *Chao* 1 were lower. At the end of the bloom, Shannon diversity (***H'***) was highest with 4.43, bacterial Simpson diversity (1/***D***) was higher on Sep. 30 (Table 3). A similar study conducted by Yang et al. [10] suggested that the water bacterial community ACE estimator ranged from 369 to 477, in addition, the highest *Chao* 1 was observed in August with 497 in the Jinpen drinking water reservoir.

To assess the water fungal community diversity, we chose to employ species richness estimates (*Chao*1, abundance- based coverage estimator, ACE) and diversity indices (Shannon, Simpson). As shown in Table 4, at the beginning of algal bloom, the ACE and *Chao* 1 indices were lowest, however, after algal bloom, the highest ACE and *Chao* 1 indices were 342 and 348, respectively. However, the Simpson diversity (1/*D*) index remained at a low level during outbreak of algal bloom on Sep. 1, and remarkably increased at the end of bloom decline period (Table 4).

To further explore the shifts of water bacterial and fungal community compositions, 16S rRNA and ITS sequences from each sample were classified with BLAST and Ribosomal Database Project (RDP) classifier, UNITE database (http://unite.ut.ee) [22,25].

**Figure 3:**
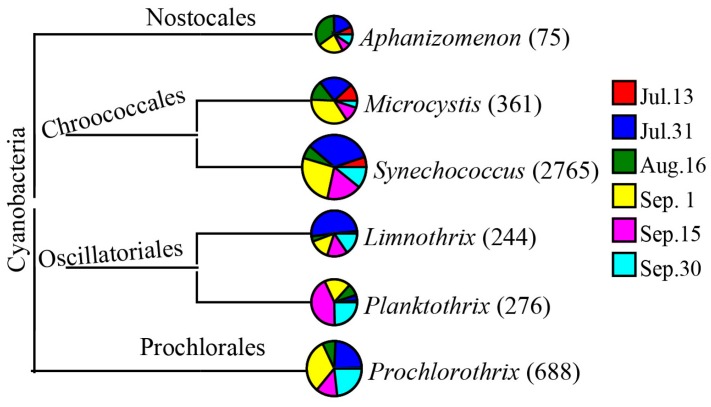
Taxonomic classification of cyanobacterial community in Zhoucun drinking water reservoir from July to September, 2012. Number in the bracket represents OTUs number.

**Figure 4:**
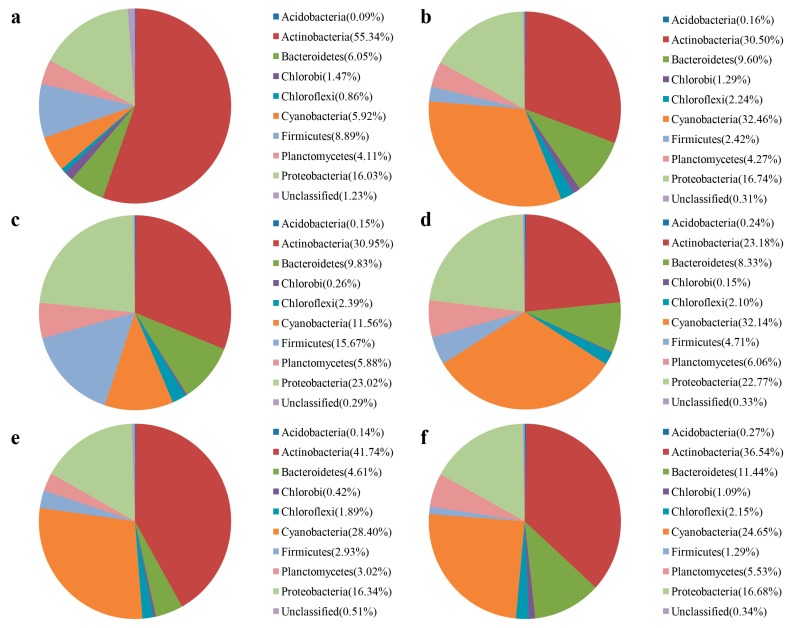
Taxonomic classification of water bacterial community in Zhoucun drinking water reservoir during algal bloom outbreak and decline from July to September, 2012. Phylum levels using the Ribosomal Database Project classifier. a, b, c, d, e, f represent Jul. 13, Jul. 31, Aug. 16, Sep. 1, Sep. 15, Sep. 30 sampling date, respectively.

Based on the Illumina Miseq sequence data, as shown in Figure 3, the dominant cyanobacteria were *Synechococcus* (accounting for 62.71% of the total effective sequences), *Prochlorothrix* (15.60%), and *Microcystis* (8.19%). This is consistent with microscopic examination results. For bacterial community, as shown in Figure 4, dominant phyla were *Actinobacteria*, *Cyanobacteria*, *Proteobacteria*, *Bacteroidetes*, and *Firmicutes*.

However, the water bacterial community structure changed through time, with different dominant species before (Figure 4a−c), during (Figure 4d) and after (Figure 4e,f) blooms. Before the outbreak of bloom, *Actinobacteria* decreased steadily from 55.34% to 30.50%. During the bloom, Cyanobacteria was 32.14%, *Actinobacteria* was 23.18%, *Bacteroidetes* was 8.33%, *Firmicutes* was 4.71%. *Proteobacteria* increased during the outbreak of bloom. The abundance of *Proteobacteria* was positively correlated with algal cell concentration (*p* < 0.05). The highest ACE index was also found in Sep. 15. Certain groups of bacteria (e.g., *Proteobacteria*) tend to increase in abundance during blooms. Consistent with our study, research conducted by Xue et al. [32] reported that cyanobacterial blooms triggered a significant increase in abundance of anaerobic ammonium oxidizing (anammox) bacteria in a subtropical reservoir, and that as a result the bacterial community was different during the bloom period compared with non-bloom periods. Meanwhile, Luria et al. [29] found that Flavobacteria and Rhodobacteraceae performed better during non-bloom conditions. *Proteobacteria*, *Bacteriodetes*, *Actinobacteria* are widely distributed taxa and are also found in Lakes Erie, Michigan and Huron, located in the eastern of North America [33].

**Figure 5:**
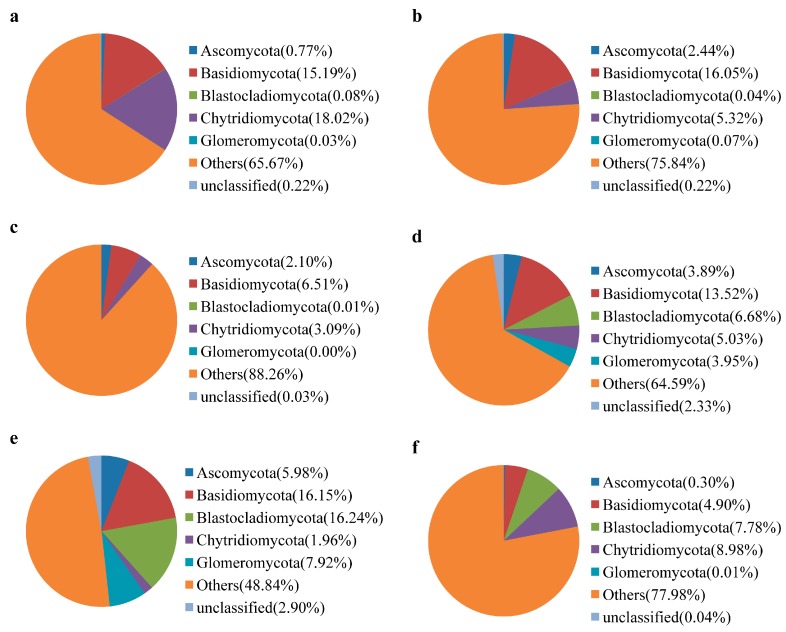
Taxonomic classification of water fungal community in Zhoucun drinking water reservoir during algal bloom outbreak and decline from July to September, 2012. Phylum levels using the Ribosomal Database Project classifier. a, b, c, d, e, f represent Jul. 13, Jul. 31, Aug. 16, Sep. 1, Sep. 15, Sep. 30 sampling date, respectively.

Water fungal diversity plays an important role in reservoir ecosystems because fungi can degrade organic matter. To the best of our knowledge, this study represents one of only a few studies to examine fungal community in drinking water reservoirs. At the phylum level, the water fungal community changed during the outbreak and decline periods of algal bloom in the Zhoucun drinking water reservoir. Figure 5 provides an overview of the water fungal community structure. Before outbreak of the bloom, the dominant phylum was Chytridiomycota at 18.02% of the community, whereas, Basidiomycota was dominant during the bloom outbreak at 13.52% (Figure 5d). During the decline period, the dominant phylum was Blastocladiomycota at 16.24% (Figure 5e). At the end of decline on Sep. 30, Chytridiomycota (8.98%) and Blastocladiomycota (7.78%) were dominant. Meanwhile, the majority (approximately 80%) of the fungal sequences detected in Zhoucun drinking water reservoir belong to unclassified phylum. It might be that Zhoucun drinking water reservoir harbors a distinct fungal community composition.

**Figure 6:**
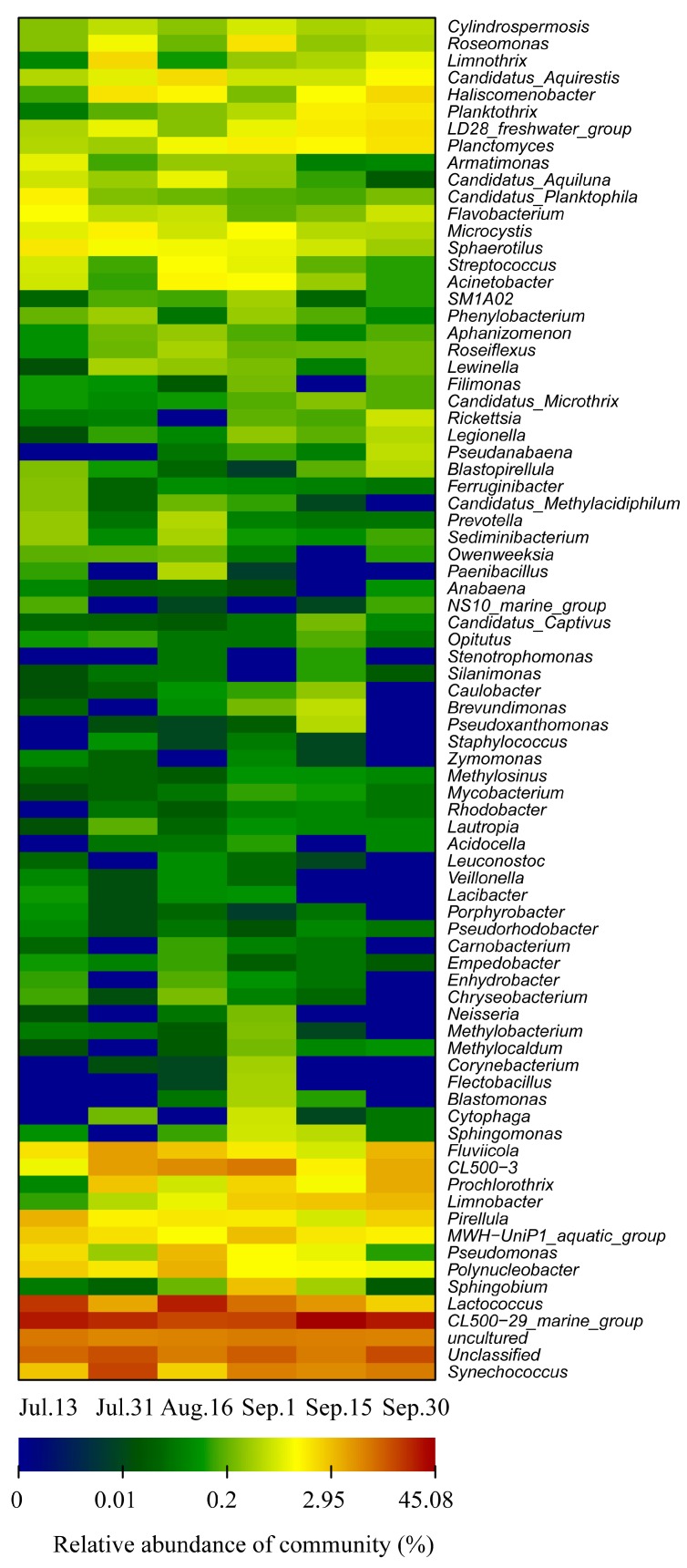
Heat map showing 80 representative predominant 16S rRNA gene based sequence classified at the genus level in Zhoucun drinking water reservoir during algal bloom outbreak and decline from July to September, 2012. Blue colors indicate lower abundance, and red colors indicate higher abundance.

As shown in Figure 6, a heat map fingerprint was drawn with the top 80 most abundant bacterial species at the genus level. The water bacterial communities before, during and after bloom clearly differed. *Pirellula* sp. and *Lactococcus* sp. were the most abundant taxa on Jul. 13, while the dominant genus on Aug. 16 was *Lactococcus* sp. The dominant genus were *Acinetobacter* sp., *Limnobacter* sp., *Roseomonas* sp., *Synechococcus* sp., and *Sphingobium* sp. during the bloom outbreak on Sep. 1. As shown by the heat map patterns, the water bacterial compositions changed significantly from the beginning of the outbreak to the decline of the bloom. Similar studies also demonstrated that phytoplankton blooms affect the composition of bacterial communities. This may be driven be the large amount of nitrogen and organic carbon released during the blooms’ decomposition, which can be used by the bacterial community [34]. Temporal changes in nutrient and carbon availability may also drive the pronounced successions of distinct taxa within the water bacterial community. Previous studies suggested that *Acinetobacte* sp. was also detected in drinking water reservoirs [11]. Interestingly, *Roseomonas* sp. strain was isolated from the drinking water distribution system of Seville (Spain) [35]. From late June to mid-August, *Synechococcus* sp. was the dominant species in two recreational lakes in South Germany during cyanobacterial blooms [7]. Previously, Rinta-Kanto et al. [34] observed that SAR11 and SAR86 groups were decreased during algal blooms, and *Flavobacteria* SAR324 were increased 350% between bloom and control microcosms dominated by diatoms [34]. Teeling et al. [36] found that Roseobacter clade, a member of Alphaproteobacteria, dominated in pre-bloom communities and changed following the bloom. However, *Formosa* spp. dominated in phytoplankton bloom. *Reinekea* spp. had highest abundances during algal decay [36]. Interestingly, in this study, *Roseomonas* sp. was an abundant taxa observed during blooms instead of pre-bloom. Both the Cytophaga-Flavobacteria lineages of Bacteroidetes and Betaproteobacteria showed similar growth trends during and after the phytoplankton bloom [37]. The above mentioned bacterial communities, such as *Acinetobacter* sp., *Limnobacter* sp., *Roseomonas* sp., *Synechococcus* sp., and *Sphingobium* sp. commonly exist in lakes or reservoirs [11,37]. Although there are differences in bacterial communities between lakes and reservoirs, the Alphaproteobacteria, Gamma- proteobacteria and Bacteroidetes are the main bacterial community components in the phase of phytoplankton bloom [4,5,6,7,8,9]. Interestingly, *Microcystis* sp. and *Synechococcus* sp. were also observed in heat map fingerprints. Abundance of *Synechococcus* sp. was 7.66 times higher than that of *Microcystis* sp. Abundance of *Microcystis* sp. increased before bloom, this may be the result of an increase in available ammonia on Aug. 16.

**Figure 7:**
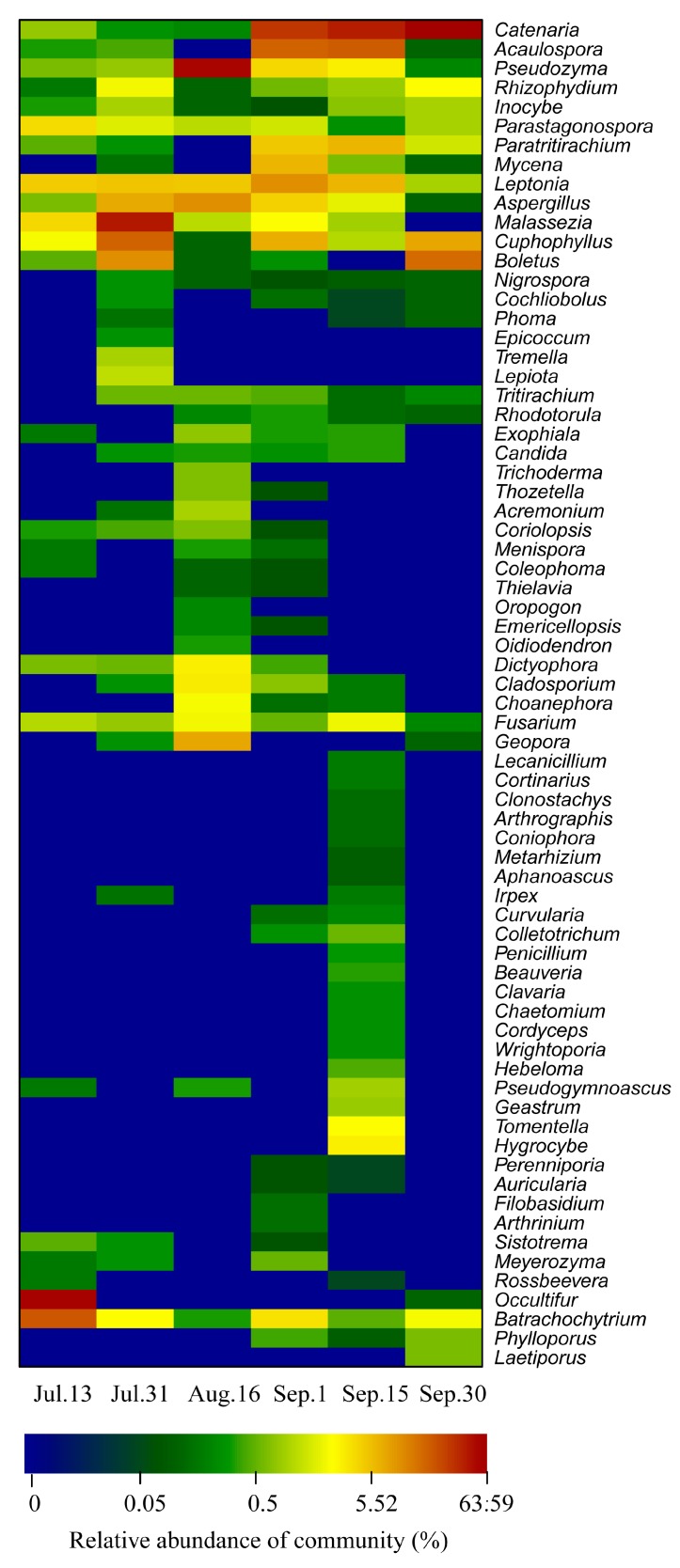
Heat map showing 70 representative predominant fungal ITS gene based sequence classified at the genus level in Zhoucun drinking water reservoir during algal bloom outbreak and decline from July to September, 2012. Blue colors indicate lower abundance, and red colors indicate higher abundance.

Concomitantly, at the genus level, a heat map profile was drawn with top 70 abundant fungal species, showing water fungal communities dynamic during the outbreaks and decline of phytoplankton blooms in the Zhoucun drinking water reservoir (Figure 7). On Jul. 13, the predominant fungal groups were affiliated with *Occultifur* sp. (56%) and *Batrachochytrium* sp. (21%). During the outbreak of bloom on Sep. 1, the dominant genus were *Catenaria* sp. (29.7%) and *Acaulospora* sp. (17.6%), whereas *Catenaria* sp. (41%), *Paratritirachium* sp. (6.7%) and *Boletus* sp. (16.0%) were dominant during the decline periods on Sep. 15 and 30 (Figure 7). In reservoir ecosystems, organic matter (OC) quality structures the abundance of water fungal community [38].

**Figure 8:**
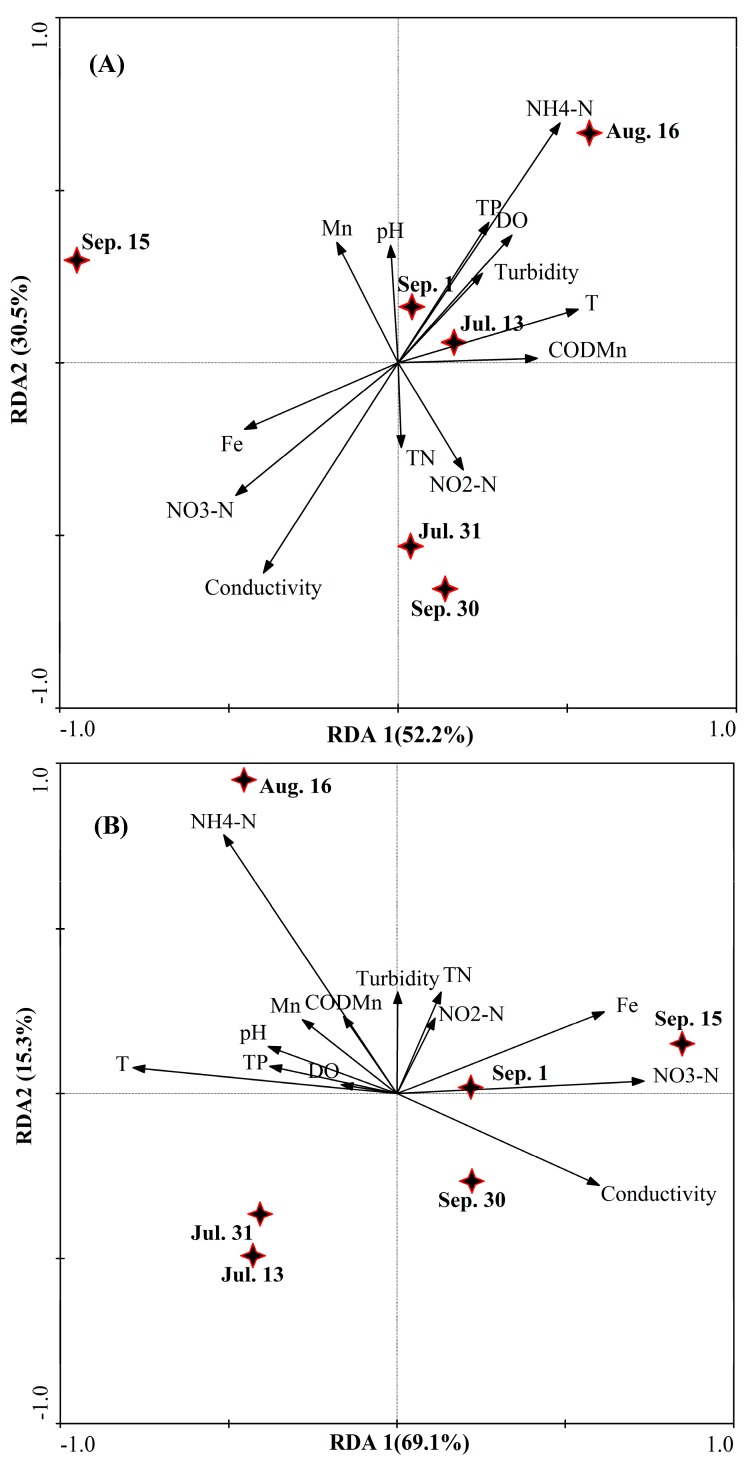
Redundancy analysis (RDA) of water bacterial (**A**) and fungal (**B**) communities in Zhoucun drinking water reservoir during algal bloom outbreak and decline from July to September, 2012. Stars represent sampling points including Jul. 13, Jul. 31, Aug. 16, Sep. 1, Sep. 15, and Sep. 30. For water bacterial community, RDA1 explained 52.2 %, and RDA2 explained 30.5% of the total variance. For water fungal community, RDA1 explained 69.1%, and RDA2 explained 15.3% of the total variance. The primary factors for the variables of the water quality data are represented by arrows (DO=dissolved oxygen; TN = total nitrogen; TP=total phosphorus; COD_Mn_ = permanganate index).

To evaluate the relationship between water quality and the structure of bacterial and fungal communities during blooms, multivariate analysis was performed. Redundancy analysis (RDA) indicated distinct water bacterial and fungal communities during the outbreak and decline of blooms in Zhoucun drinking water reservoir. As shown in Figure 8, a biplot of the water quality variables and genus-level of microbial community structure was generated using a redundancy analysis (RDA) model and was employed to investigate the relationship between water quality parameters and the structure compositions of bacterial and fungal community based on Illunina MiSeq sequencing data.

The RDA performed on Illunina MiSeq sequencing showed that RDA1 and RDA2 could explain 82.7 % of the total variation (Figure 8A). RDA also showed that the structure of water bacterial communities before, during and after blooms was significantly correlated to conductivity (*p* < 0.05, Monte Carlo permutation), and ammonia nitrogen (*p* < 0.01). RDA reveals that water bacterial community (after the bloom on Sep. 15) in the second quadrant in the plot (Figure 8A). Water temperature and nitrate nitrogen were significant factors in explaining the variation in water bacterial community along RDA 1 (*p* < 0.05). The RDA model was also applied to identify the water quality parameters influencing the fungal community compositions. As shown in Figure 8B, RDA1 and RDA2 explained 84.4% of the total variance. Water temperature, conductivity, Fe and ammonia nitrogen significantly influenced on water fungal communities during outbreak and decline of blooms from Jul. 13 to Sep. 30 (*p* < 0.01, *p* < 0.01, *p* < 0.05, *p* < 0.01). RDA reveals that water fungal community (before the bloom on Jul. 13, Jul. 31, and Aug. 16) in the second and third quadrants of the plot (Figure 8B).

In freshwater ecosystems, the water fungal community can drive the nutrient cycling through processes, such as decomposition. Although the microbial community patterns during phytoplankton blooms have been well studied [4,5,6,7,8,9], the literature on the fungal community in drinking water reservoir ecosystems is still very limited. Sediment fungal community associated with different drinking water reservoirs were previously described by quantitative PCR and 454 Pyrosequencing [12], and revealed that the fungal communities were dominated by OTUs belonging to the Chytridiomycota, including the Chytridiomycetes, Monoblepharidomycetes, and Blastocladiomycetes. The dynamics of water fungal community during a dinoflagellate (*Noctiluca scintillans*) bloom has also been investigated [39], and it was found that Ascomycota, Mucoromycotina, Chytridiomycota, and Basidiomycota were the predominant fungal types. N and P were found to significantly influence the fungal population structure during *N. scintillans* bloom. In oligo-mesotrophic lakes, Lefèvre at el. [40] found 19% of the sequences belonged to the parasitic *Chytridiomycota*, especially in spring and summer. In the present work, *Chytridiomycota* was reduced from 18.02% in Jul. 13 to 1.96% in Sep. 15. *Chytridiomycota* can produce microscopic free-swimming spores and parasitic chytrids which have an ability to infect various hosts, including diatoms and filamentous species. *Chytridiomycota* is thought to also play an important role in the aquatic environment food chain [41]. The microcosms experiment constructed by Zhao et al. [31], found that the water bacterial community diversity indices such as the Shannon diversity index and evenness were increased during the decomposition of *Microcystis* biomass. Interestingly, in our previous study, we observed that the water fungal community of the Zhoucun drinking water reservoir was different from that of the sediment [11]. *Elaphomyces* (20.00%) and *Rhizophydium* (13.84%) dominated in sediment, while *Occultifur* sp., *Catenaria* sp. and *Acaulospora* sp. were dominant in water. The unassigned water fungi proportion in Zhoucun drinking water reservoir was up to 88.26%. The reason for this inconsistency was likely that the different reservoir and water quality lead to the different results. Recently, Zhang et al. [42] used 454-pyrosequencing to study the shifts of denitrifying bacterial community diversity during a cyanobacterial bloom in a eutrophic shallow lake in China, and revealed that the community structure of denitrifying bacteria changed with the increase of algal cell density.

In the past few decades, understanding of the relationship between water/sediment microbial composition and cyanobacterial blooms has revealed complex interactions between blooms and microbial diversity [28]. Our work leaves open a number of interesting issues related to the relationship between algal blooms and water microbial communities. In this work, genus level morphological identification of cyanobacteria showed the presence of *Synechococcus*, *Microcystis*, and *Prochlorothrix*. More cyanobacterial species such as *Synechococcus*, *Prochlorothrix*, *Microcystis*, *Aphanizomenon*, *Limnothrix*, and *Planktothrix,* were successfully detected using Illumina Miseq sequencing, thus highlighting the power of using a high throughput sequencing technique in exploring cyanobacteria. The same major cyanobacterial species were observed with both techniques, but relative species abundance estimates differed. Similarly, according to morphological identification, Parulekar et al. [6] observed that *Aphanizomenon*, *Microcystis*, *Chroococcous* and *Woronichinia* dominated the cyanobacterial community throughout the season in Akersvannet Lake, South Norway. Illumina sequencing revealed the bacterial community was dominated by *Cyanobacteria, Proteobacteria, Actinobacteria*, and *Verrucomicrobia*.

Algal cells can secrete dissolved organic matter (DOM), which will significantly affect the structure of bloom-associated bacterial communities [29]. Meanwhile, *Microcystis* produce microcystin that can shape the structure of bacterial communities, and the response of the water microbial community to toxic and non-toxic blooms is different [28]. Our results contribute toward a greater understanding of the dynamics of bacterial and fungal communities during the outbreak and decline of algal blooms in drinking water reservoirs. It would be of interest to focus future studies on identifying the drivers of shifts in functional water/sediment microbes (cable bacteria, denitrifying bacteria, manganese oxidizing bacteria and sediment anammox bacteria) structures due to algal blooms, and to elucidate the impact of the combined influence on reservoir water quality.

## 4. Conclusions

This study contributes the first conclusive overview of the dynamics of bacterial and fungal communities associated with algal blooms in a drinking water reservoir using 16S rRNA combined with ITS regions targeted amplicon sequencing. We found distinctly different degrees of temporal variation between major water quality (e.g., pH, TN, TP, COD_Mn_, Fe and Mn) during the bloom decline period compared with bloom outbreak periods. During the algal bloom, the maximum algal biomass and cell concentrations measured were 51.7 mg/L and 1.9 × 10^8^ cell/L, respectively. A total of 2,297 and 1,346 OTUs were generated for water bacterial and fungal communities, defined at 97% identity of the 16 S rRNA and ITS genes. Shannon diversity (*H'*) of the fungal community was remarkably increased when algal blooms occurred. Redundancy analysis (RDA) suggested that the water bacterial community structure was significantly correlated to conductivity and ammonia nitrogen. Meanwhile, water temperature, Fe, and ammonia nitrogen were significantly correlated with water fungal communities during the outbreak and decline of blooms. Our exploration of water bacterial and fungal diversity following different algal bloom patterns provides novel insights on the potential role of the water microbes possibly driving outbreak and decline processes thus can be utilized to develop sustainable bloom management strategies in reservoir ecosystems.

## Figures and Tables

**Table 1:** Specific primer sets of water bacteria and fungi, and PCR program process.

Water Microbe	Amplified Nuclear Ribosomal Regions	Primer Sets (from 5' to 3')	PCR Program
Bacteria [19,20]	16S rRNA V4-V5 region	515F: GTGCCAGCMGCCGCGGTAA 907R: CCGTCAATTCCTTTGAGTTT	95°C for 2 min, 30 cycles of 95°C for 30 s, 55°C for 30 s, 72°C for 30 s, with a final extension at 72°C for 5 min
Fungi [21]	Internal transcribed spacer-1 (ITS1) region	1737F:GGAAGTAAAAGTCGTAACAAGG 2043R:GCTGCGTTCTTCATCGATGC	95°C for 1 min, 30 cycles of 95°C for 10 s, 55°C for 30 s, 72°C for 30 s, with a final extension at 72°C for 5 min

**Table 2:** Water quality parameters of Zhoucun drinking water reservoir during algal bloom outbreak and decline from July to September, 2012.

Sampling date	T (℃)	DO (mg/L)	Turbidity (NTU)	Conductivity (S/cm)	pH	COD_Mn_ (mg/L)	TN (mg/L)	NO_3_^−^-N (mg/L)	NO_2_^−^-N (mg/L)	NH_4_^+^-N (mg/L)	TP (mg/L)	Fe (mg/L)	Mn (mg/L)
Jul. 13	28 ± 0.06	12.5 ± 0.12	7.3 ± 0.2	271 ± 1.7	8.38 ± 0.01	4.46 ± 0.28	1.80 ± 0.02	0.91 ± 0.01	0.03 ± 0.0	0.34 ± 0.03	0.07 ± 0.0	0.05 ± 0.0	0.08 ± 0.0
Jul. 31	31 ± 0.06	7.3 ± 0.3	14.7 ± 0.1	286 ± 3.5	8.65 ± 0.02	7.52 ± 0.02	2.6 ± 0.03	1.44 ± 0.03	0.15 ± 0.0	0.45 ± 0.03	0.06 ± 0.0	0.11 ± 0.0	0.09 ± 0.0
Aug. 16	29 ± 0.0	10.2 ± 0.11	18.2 ± 0.06	275 ± 2.2	8.45 ± 0.01	7.4 ± 0.07	2.42 ± 0.06	1.28 ± 0.03	0.13 ± 0.0	0.78 ± 0.05	0.06 ± 0.0	0.12 ± 0.0	0.09 ± 0.0
Sep. 1	28.3 ± 0.1	16.1 ± 0.2	33.1 ± 0.06	276 ± 1.4	8.4 ± 0.05	10.11 ± 0.11	2.4 ± 0.06	1.43 ± 0.07	0.16 ± 0.0	0.44 ± 0.02	0.08 ± 0.0	0.08 ± 0.0	0.06 ± 0.0
Sep. 15	23.2 ± 0.1	7.27 ± 0.06	9.3 ± 0.01	306 ± 4.2	8.4 ± 0.03	4.52 ± 0.26	2.31 ± 0.11	1.77 ± 0.12	0.09 ± 0.0	0.32 ± 0.01	0.05 ± 0.0	0.17 ± 0.0	0.09 ± 0.0
Sep. 30	22.5 ± 0.2	7.87 ± 0.06	10.2 ± 0.02	332 ± 3.8	7.9 ± 0.0	6.29 ± 0.16	2.42 ± 0.11	1.9 ± 0.08	0.15 ± 0.0	0.16 ± 0.0	0.03 ± 0.0	0.17 ± 0.0	0.05 ± 0.0
ANOVA	**	**	***	*	*	***	*	*	**	**	**	***	*

T: water temperature. DO: dissolved oxygen. TN: total nitrogen. TP: total phosphorus. COD_Mn_: permanganate index. Data showed as means ± standard deviations (*n* = 3). * *p* < 0.05; ** *p* < 0.01; *** *p* < 0.001 represent statistical significance using One way ANOVA.

**Table 3:** Water bacterial community diversity indices measured based on the Illumina MiSeq sequencing data of Zhoucun drinking water reservoir during algal bloom outbreak and decline from July to September, 2012.

Sampling date	ACE	*Chao*1	Shannon diversity (*H'*)	Simpson diversity (1/*D*)
Jul. 13	383 (363,415)	386 (361,433)	3.58 (3.55,3.62)	11 (11,11)
Jul. 31	409 (386,445)	405 (380,451)	4.19 (4.16,4.22)	25 (25,25)
Aug. 16	463 (443,495)	458 (436,498)	3.99 (3.96,4.03)	16 (16,16)
Sep. 1	507 (493,531)	514 (494,552)	4.33 (4.31,4.35)	33 (33,33)
Sep. 15	459 (436,493)	454 (429,497)	4.18 (4.15,4.21)	25 (25,25)
Sep. 30	401 (381,435)	399 (375,442)	4.43 (4.39,4.46)	33 (33,33)

ACE: Abundance-based coverage estimators.

**Table 4:** Water fungal community diversity indices measured based on the Illumina MiSeq sequencing data of Zhoucun drinking water reservoir during algal bloom outbreak and decline from July to September, 2012.

Sampling date	ACE	*Chao* 1	Shannon diversity (*H'*)	Simpson diversity (1/*D*)
Jul. 13	233 (219,259)	238 (219,276)	3.48 (3.43,3.52)	12 (14,12)
Jul. 31	324 (289,374)	259 (233,307)	3.51 (3.46,3.56)	14 (14,14)
Aug. 16	243 (218,286)	236 (210,288)	1.29 (1.26,1.32)	1.61 (1.61,1.62)
Sep. 1	327 (309,357)	322 (303,358)	4.11 (4.07,4.15)	25 (25,25)
Sep. 15	342 (322,374)	348 (322,396)	4.04 (4.00,4.09)	20 (20, 20)
Sep. 30	256 (228,303)	247 (218,302)	2.26 (2.21,2.31)	3.03 (3.01,3.04)

ACE: Abundance-based coverage estimators.
